# Glby, Encoded by *MAB_3167c*, Is Required for *In Vivo* Growth of Mycobacteroides abscessus and Exhibits Mild β-Lactamase Activity

**DOI:** 10.1128/jb.00046-22

**Published:** 2022-04-05

**Authors:** Christos Galanis, Emily C. Maggioncalda, Pankaj Kumar, Gyanu Lamichhane

**Affiliations:** a Center for Tuberculosis Research, Department of Medicine, School of Medicine, Johns Hopkins University School of Medicine, Baltimore, Maryland, USA; Geisel School of Medicine at Dartmouth

**Keywords:** MAB_3167c, Glby, β-lactamase, *Mycobacterium abscessus*, *Mycobacteroides abscessus*

## Abstract

Mycobacteroides abscessus (*Mab*; also known as Mycobacterium abscessus) is an emerging opportunistic pathogen. Patients with structural lung conditions such as bronchiectasis, cystic fibrosis, and chronic obstructive pulmonary disease are at high risk of developing pulmonary *Mab* disease. This disease is often chronic as the current treatment regimens are sub-efficacious. Here, we characterize the phenotype of a *Mab* strain lacking the *MAB_3167c* locus, which encodes a protein hereafter referred to as Glby. We demonstrate that the loss of Glby impairs normal planktonic growth in liquid broth, results in longer average cell length, and a melding of surfaces between cells. Glby also exhibits a mild β-lactamase activity. We also present evidence that amino acid substitutions that potentially alter Glby function are not favored. Lastly, we demonstrate that, in a mouse model of pulmonary *Mab* infection, the mutant lacking Glby was unable to proliferate, gradually cleared, and was undetectable after 3 weeks. These data suggest that an agent that inhibits Glby *in vivo* may be an efficacious treatment against *Mab* disease.

**IMPORTANCE** Mycobacteroides abscessus can cause chronic pulmonary infections requiring administration of multiple antibiotics, still resulting in a low cure rate. The incidence of M. abscessus disease is increasing in the United States and the developed regions of the world. We show for the first time that a protein, Glby, affects growth of this bacterium. Using a mouse model of lung M. abscessus disease, we demonstrate that Glby is required for this bacterium to cause disease.

## INTRODUCTION

Mycobacteroides abscessus (*Mab*) is a rapidly growing non-tuberculous mycobacterium. It is an opportunistic pathogen that can cause chronic infections with incidences predominantly in patients with structural lung conditions such as bronchiectasis, cystic fibrosis, and chronic obstructive pulmonary disease (1–3). In cystic fibrosis patients, *Mab* is one of the most frequently isolated non-tuberculous mycobacteria (4–7). Additionally, *Mab* soft tissue infections in cosmetic surgery patients have also been reported (8, 9).

*Mab* is considered an emerging pathogen whose incidence in the U.S. is rising (10). *Mab* infection can be acquired from the environment, from infected individuals or via fomite intermediates (11–16). A recent report declared *Mab* “an environmental bacterium turned clinical nightmare” (17) citing the following reasons: (i) *Mab* disease is associated with rapid lung function decline and is often incurable (5, 18, 19); (ii) there are no FDA approved drugs to treat *Mab* disease, and the cure rate with the current treatment regimens, which are based on repurposed antibiotics that need to be taken daily for at least 1 year, is only 30% to 50% (20); and (iii) *Mab* is intrinsically resistant to most antibiotics used today to treat *Mab* disease (21, 22). To make matters worse, a number of antibiotics from this limited selection are associated with frequent toxicities (23). Current guidelines for treating *Mab* disease are based on clinical experience on repurposing antibiotics, as regimens informed from a clinical trial have yet to be developed (24, 25). Despite the increasing incidence of this disease, there are critical knowledge gaps in the fundamental biology of this unique microbe, which, as emerging evidence suggests, is distinct in many ways from the mycobacteria genus.

A fundamental component of bacterial cell walls is its peptidoglycan. It is the exoskeleton of bacterial cells and is required for their viability, cellular growth, and division. A major class of proteins that is involved in the final step of peptidoglycan synthesis in bacteria are D,D-transpeptidases that catalyze formation of transpeptide linkages between the fourth amino acid of one stem peptide and the third amino acid of another stem peptide (26, 27). This class of proteins is also commonly referred to as penicillin binding protein (PBP) as they were discovered as proteins that bind to penicillins. Subsequent studies revealed that this class includes proteins with D,D-transpeptidase and/or transglycosylase and d,d-carboxypeptidase activities (26). Therefore, the term PBP is a historical relic and does not capture the activities most relevant to cell wall biosynthesis and metabolism and only signifies their ability to bind to penicillins. In this study, only those proteins in *Mab* with homology to Mycobacterium tuberculosis proteins with D,D-transpeptidase, transglycosylase and d,d-carboxypeptidase activity were of interest. Additionally, the pathway for peptidoglycan synthesis is enriched in genes essential for bacterial viability, and mimics of metabolites generated in this pathway exhibit antimycobacterial activity (28). The relevance of these proteins in *Mab* to its viability, virulence, and cellular physiology has not yet been directly described.

Although the core genome content of *Mab* has a deep ancestral branching point off from other mycobacteria spp. (29), the well-characterized genome of M. tuberculosis has many conserved features when compared with *Mab*. The M. tuberculosis genome encodes for 10 putative D,D-transpeptidases/transglycosylases and d,d-carboxypeptidases (30–32). The D,D-transpeptidases are involved in synthesis of peptidoglycan whereas the d,d-carboxypeptidases catalyze removal of terminal amino acid from peptidoglycan sidechains (33). The following five M. tuberculosis proteins belong to the D,D-transpeptidases class: PbpA (Rv0016c), PbpB (Rv2163c), Pbp-lipo (Rv2864c), PonA1 (Rv0050), and PonA2 (Rv3682). The amino acid sequences of these proteins were used as a template to identify five homologs in *Mab*, of which the strain lacking *MAB_3167c* demonstrated a distinct phenotype in liquid culture, and therefore is the subject of this study. We note that the sequence homologs of M. tuberculosis D,D-transpeptidases in *Mab* are only presumed to be putative D,D-transpeptidases. In reference to the “globular” appearance of this mutant in culture, and implications of this phenotype to the physiology of *Mab* which is described below, *MAB_3167c* is hereafter referred to as *glby* (Globby). We investigated the relevance of *glby* to *Mab* growth in standard laboratory media, cellular morphology, enzymatic activity against a β-lactam reporter, and viability and growth in a mouse model of pulmonary *Mab* disease.

## RESULTS

### Glby’s amino acid sequence is highly conserved in *Mab* clinical isolates.

We used the five proteins of M. tuberculosis belonging to the D,D-transpeptidase class, PbpA (Rv0016c), PbpB (Rv2163c), Pbp-lipo (Rv2864c), PonA1 (Rv0050), and PonA2 (Rv3682), as templates to identify homologs in *Mab*. They are *MAB_0035c*, *MAB_0408c*, *MAB_2000*, *MAB_3167c*, and *MAB_4901c*, respectively. Multiple attempts to generate *Mab* strains with deletion of *MAB_0035c* and *MAB_2000* did not yield any colonies. We were able to generate *Mab* strains lacking *MAB_0408c*, *MAB_3167c*, and *MAB_4901c.* Growth and colony morphology phenotypes of *Mab* lacking *MAB_0408c* or *MAB_4901c* were unremarkable compared with the parent strain. *Mab* lacking *MAB_3167c* exhibited a distinct growth phenotype (described below) and therefore was considered for further study.

**FIG 1:**
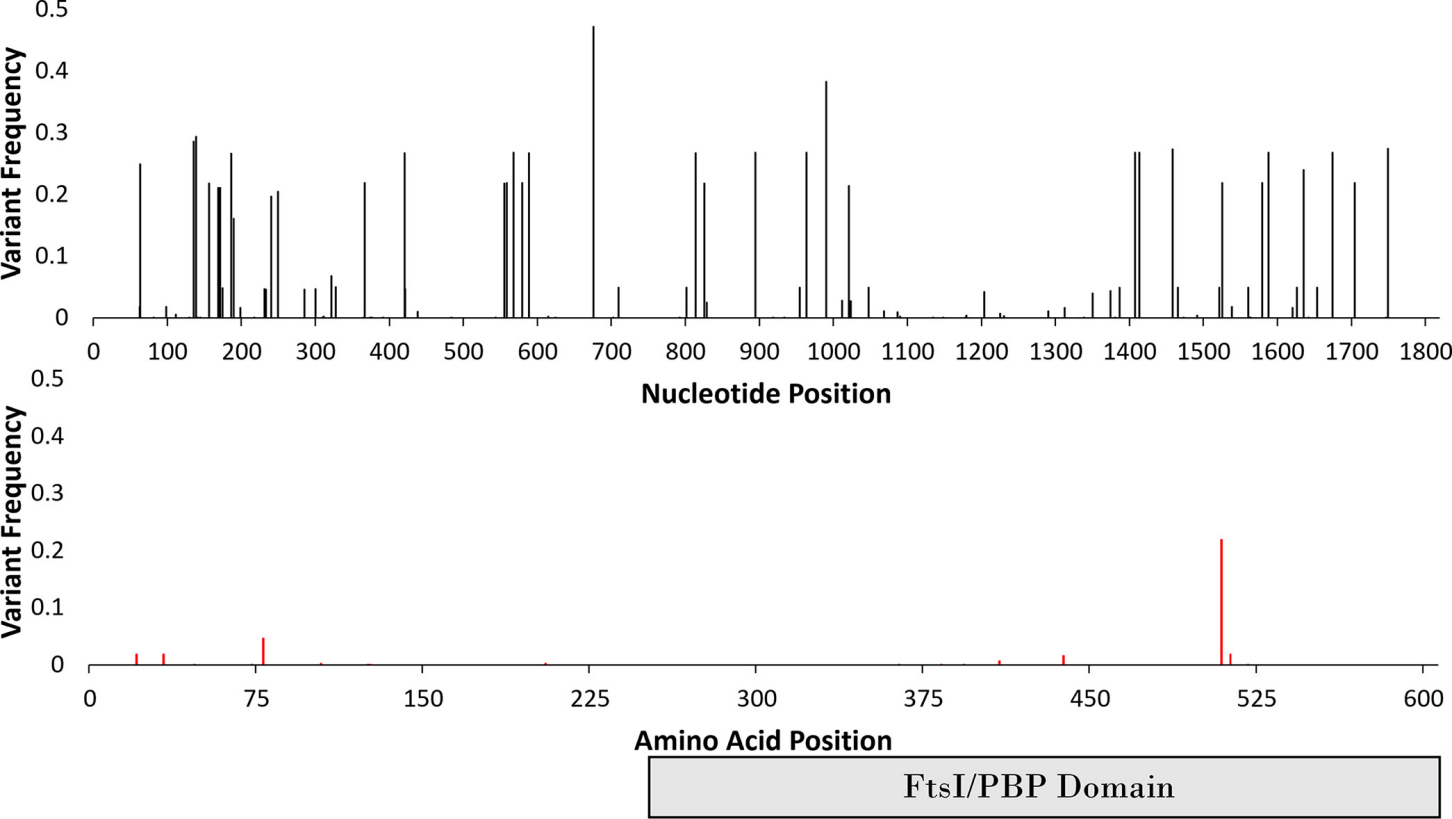
Frequencies of mutations in *glby* in 1,046 M. abscessus clinical isolates. Top-panel: frequencies of all single nucleotide polymorphisms. Bottom-panel: frequencies of SNPs that resulted in amino acid substitutions in Glby. Each bar represents mutation at the specified location. FtsI/PBP represents the predicted functional/catalytic domain characteristic of PBPs.

*Mab* isolates recovered from patients display significant genomic heterogeneity and differences in antibacterial susceptibility profiles, indicating that most of the clinical isolates represent a non-clonal collection (34). It has been previously established in multiple genome wide association studies across different species of bacteria and different organisms that regions containing vital sequences for organism survival are more likely to be conserved, as mutations that significantly alter the sequence, often lead to a loss of fitness (35–37). We therefore hypothesized that if *glby* is necessary for infection, any amino acid mutations would likely be deselected for in a collection of clinical isolates. We analyzed the *glby* locus (*MAB_3167c*) in the genomes of 1,046 independent clinical isolates from across the world that are archived in the publicly accessible database PATRIC (38) and built the consensus sequence for this gene (Fig. S1). This database included origins of the isolates, which permitted analysis of genomic variations in different regions of the world (Table 1). Of the 110 SNP locations, only 18 resulted in an amino acid substitution (83.6% were silent mutations) and only one of those (1525A>**G**) was present in more than 5% of 1,046 strains (Fig. 1). This substitution results in a conservative missense mutation from an isoleucine to a valine, **I**509**V**, which was present in 240 isolates (22%) and was not endemic to a particular geographic region, suggesting independent evolution likely arising from selective pressure. For the countries that had enough representation, only Australia had a low percentage (6%) of isolates that harbor this mutation compared with the United Kingdom (22%), the United States (25%), and China (25%). The remaining countries did not have an adequate number of isolates to enable a statistically accurate representation of isolates that harbor this mutation. The second most prevalent amino acid substitution (49 isolates or 4.6% of 1,046 isolates) is also a conservative missense mutation, A78V, resulting from 233C>**T**. Because the most prevalent amino acid substitutions (I509V and A78V) are conservative substitutions that are considered to produce little or no change in protein function, these results demonstrate that there is evidence of selective pressure *against* changes in the amino acid sequence that may compromise Glby function. To further investigate if this selective pressure was specific to *glby* or a random event that would also affect other genes in the locus, we applied the same bioinformatic approach and assessed mutations in 10 genes upstream and 10 genes downstream of *glby* (*MAB_3157c – MAB_3177*) in the genomes of the 1,046 *Mab* clinical isolates. The frequency of mutations that resulted in amino acid substitutions was significantly lower in *glby* compared with that in the proximal region (*P*-value <0.0001) (Fig. S2, Table S1). As this difference cannot be attributed to random chance alone, this evidence suggests that there may be selective pressure against mutations with potentially deleterious effect on Glby function.

**TABLE 1:** Distribution of I509V mutation in Glby in 1,046 *Mab* clinical isolates

Country	# of isolates	# of isolates with I509V mutation (%)
United Kingdom	495	107 (22%)
China	275	68 (25%)
United States	165	42 (25%)
Australia	72	4 (6%)
Malaysia	12	8 (67%)
Denmark	9	1 (11%)
Brazil	6	5 (83%)
France	6	2 (33%)
South Korea	3	2 (67%)
Ireland	3	1 (33%)
Total	1,046	240 (22%)

### *Mab* lacking *glby* exhibits altered growth and morphology *in vitro*.

**FIG 2:**
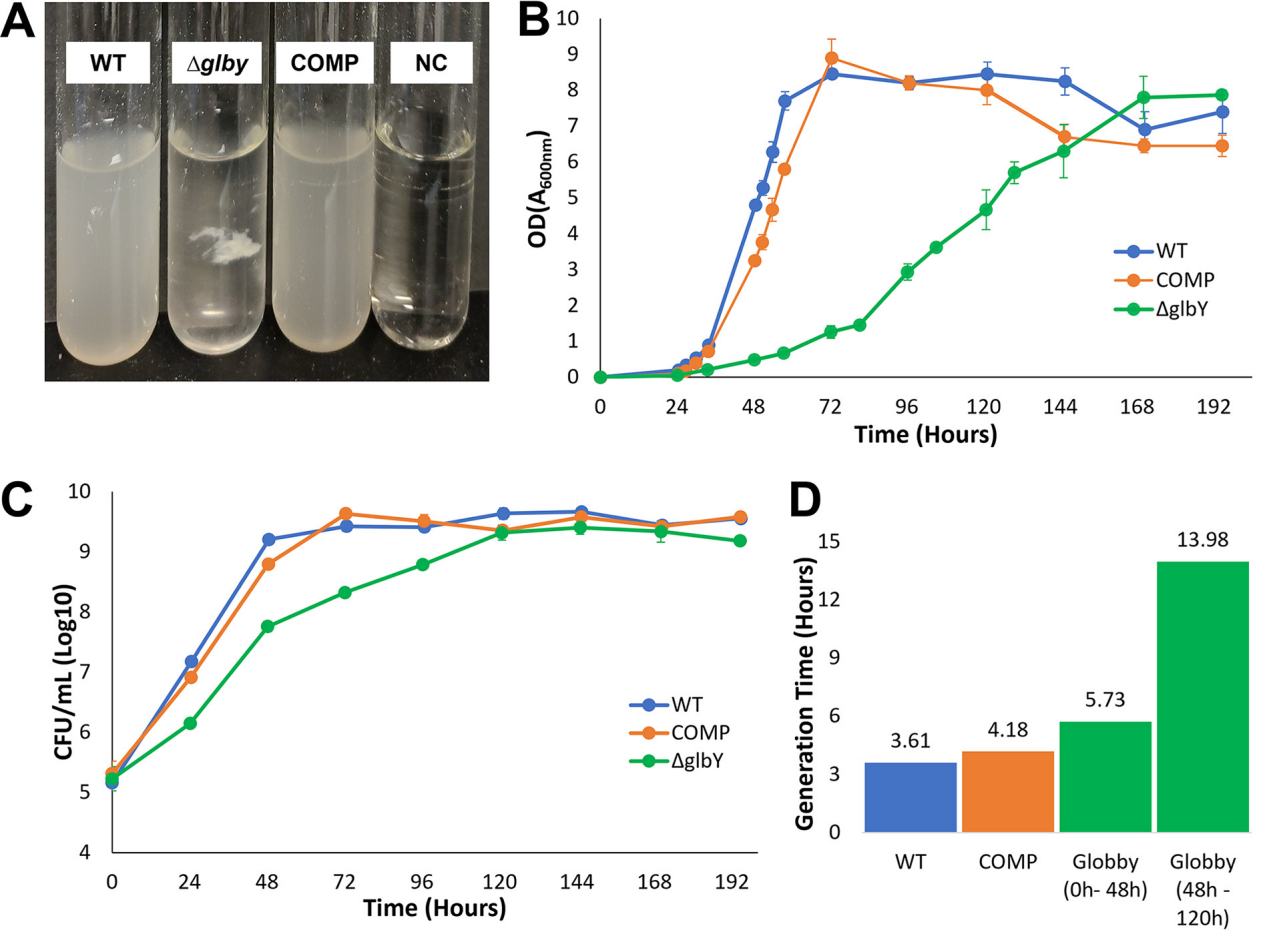
*In vitro* growth phenotypes of *Δglby*. (A) Growth of parent strain *Mab* ATCC 19977 (WT), *Δglby* and Complement (COMP) strains in Middlebrook 7H9 broth at 96 h, 37°C, constant shaking at 200 RPM. Culture media only as negative controls (NC) was also included. (B) Time course of optical densities of cultures of WT, *Δglby*, and COMP measured as absorbance at 600 nm. Each time point represents the average from four distinct replicates and no sample tube was used more than once. Error bars are standard deviation of each sample. (C) Time course of CFU of the three strains. Error bars are standard deviation of each sample. (D) Generation time (time required for a strain to double in CFU) of each strain determined from exponential phase(s) of growth. Two generation times reported for *Δglby* represent the biphasic exponential growth stages of this strain.

We used *Mab* ATCC 19977 (hereafter referred to as wild-type, WT), as the parent strain, as it is commonly used as a laboratory reference strain (39). We generated a strain lacking *glby* (hereafter referred to as *Δglby*) using a recombineering system optimized for mycobacteria (40). The genome of *Δglby* was sequenced and compared to the parent WT genome to confirm the deletion of *glby* and to verify the lack of mutations elsewhere in the genome (Fig. S3). We generated a complemented strain by inserting a copy of *glby* cloned from WT into the L5 *attB* site of the *Δglby* chromosome (*attB::pMH94apra-MAB_3167c*) (Fig. S4), using plasmid pMH94 (41) with modifications to carry an apramycin selection cassette. The genotype of this strain and the site of *glby* integration was also verified by sequencing its genome (Fig. S5). This strain is hereafter referred to as COMP. On Middlebrook 7H10 agar base, compared to WT and COMP, *Δglby* required ∼ 2 additional days to form colonies. The colony morphotype, as assessed by gross visualization of the surface architecture, namely, color and size of colonies on Middlebrook 7H10 agar, was not distinct between *Δglby* and that of the WT and COMP strains (Fig. S6). However, when grown in Middlebrook 7H9 liquid broth, *Δglby* exhibited a distinct growth morphotype. *Δglby* grew as a single globular clump during exponential phase while the broth remained clear, whereas WT and COMP grew planktonically and turned the broth turbid (Fig. 2A). Owing to the globular appearance of this strain in liquid broth, we assigned the gene locus (*MAB_3167c*) a phenotype-based annotation: *glby* (globby). The reversion of the globular growth phenotype to planktonic growth in the COMP strain demonstrates that this phenotype resulted from the lack of *glby*. Interestingly, initial growth of *Δglby* is only permissible in a globular form as ascertained from two distinct experiments. First, we subjected *Δglby* to incremental sheer forces when growing in liquid broth by altering the diameter of the culture vessel, while maintaining orbital shaking speed constant at 200 revolutions per minute (RPM). When grown in a tube with 1.5 cm diameter (14 mL culture tube, Falcon), *Δglby* grew as a large single globular clump. In a tube with 3 cm diameter (50 mL culture tube, Falcon), *Δglby* grew in several (∼ 20 to 30) smaller clumps, and, in a 150 mL flask with a 6 cm diameter, *Δglby* failed to grow for 5 days, but formed one large clump after 3 days when the shaking speed was lowered to 75 RPM. In the second experiment, we tested the hypothesis that *Δglby* requires a clumped morphotype to sustain growth in liquid broth. We dispersed a *Δglby* culture in exponential growth phase with vigorous shaking and pipetting into a planktonic suspension and incubated it at standard growth conditions of 37°C and 200 RPM orbital shaking. *Δglby* again formed globular clumps within 24 h of dispersal and the broth was clear, demonstrating that clumped growth is preferred over planktonic growth in the absence of *glby*. Based on the altered growth phenotype in liquid culture, we hypothesized that the generation time (the time it takes for CFU to double) of *Δglby* may be different compared with that of WT. To test this hypothesis, *Δglby*, WT, and COMP strains were grown under identical conditions and culture optical density (OD) and CFU were determined at regular intervals over 8 days duration (Fig. 2B and C). Importantly, because of *Δglby*’s globular morphology, OD readings would be inaccurate if not dispersed into planktonic suspension. In addition, the OD determinations would likewise be inaccurate if culture from the same tube was sampled at each successive time point as these interventions may disturb the natural course of *Δglby* growth. Therefore, four culture tubes per time point per strain were included, and each sample tube was used only once for OD and CFU determination. *Δglby* exhibited attenuated growth compared with WT and COMP in both OD and CFU determinations (Fig. 2B and C). In the OD measurement assay, *Δglby* required 168 h to attain peak OD whereas WT and COMP strains reached peak OD within 72 h. Interestingly, when *Δglby* reaches an OD of ∼4 to 5 at about 120 h of growth, which also coincides with the time point at which it reaches peak CFU density, the broth began turning turbid, as it appeared *Δglby* was sloughing off the clumps. At this time, *Δglby* existed in both clumped and suspension forms.

When the growth profile of *Δglby* was compared with WT and COMP in terms of CFU, a clear distinction emerged from the onset of growth until peak, with the largest difference happening at the 48-h time point, at which, CFU density of *Δglby* was ∼1.5 log_10_ lower compared with WT and COMP strains (Fig. 2C). While WT and COMP attained peak CFU density by 72 h, *Δglby* required an additional 48 h (120 h time point) to reach the same peak CFU. Of note, *Δglby* appeared to exhibit bi-phasic rates during the exponential stage, with a higher rate of growth between 0 h and 48 h and a reduced rate thereafter, until peaking at 120 h (Fig. 2D). In summary, these findings demonstrate that *glby* is required for growth in planktonic form and for normal growth rate; however, it is dispensable for viability or growth of *Mab*.

An unbiased query based on pairwise sequence alignment of Glby’s amino acid sequence using BLAST (42) identified a M. tuberculosis protein encoded by *Rv2864c*, with the highest similarity (64% amino acid sequence identity, 78% sequence positivity, e-value = 0). This protein is presumed to be a PBP-lipo and is predicted to encode a PBP with a putative lipoprotein attachment function in the cell wall (30, 43). Because composition of cell wall lipids determines whether various cell wall specific dyes used to probe identities of bacteria can bind and be retained, we asked whether loss of Glby affects the cell wall lipid composition using Ziehl-Neelsen (ZN) stain. This dye stains mycobacteria pink (44). If the cell wall lipid composition is affected and the ZN dye is not retained after wash with destain solution, and the counter dye methylene blue is retained, cells instead appear blue. We hypothesized that *Δglby* cells may have an altered mycolipid layer and consequently exhibit altered staining with the ZN dye. We tested this hypothesis by staining WT, *Δglby*, and COMP cells at exponential growth phase with ZN. Both WT and COMP along with the majority of *Δglby* cells appeared pink (Fig. S7). As a minority of *Δglby* cells appeared blue, we conclude that *Δglby* culture consists of two populations of cells in terms of their ZN staining properties.

To further investigate this finding, we stained WT, *Δglby*, and COMP cells with Auramine-rhodamine (AR), a fluorescent dye that is also used in microbiological identification of mycobacteria and is considered more sensitive than ZN (45). The AR compound strongly binds to mycolipids and is not efficiently washed off by the destain solution. Therefore, if the mycolipid layer is compromised, the cells will not appear fluorescent. The intensities of fluorescence were similar among the three strains, (Fig. S8), indicating that the absence of Glby does not affect the AR staining properties of *Mab*.

**FIG 3:**
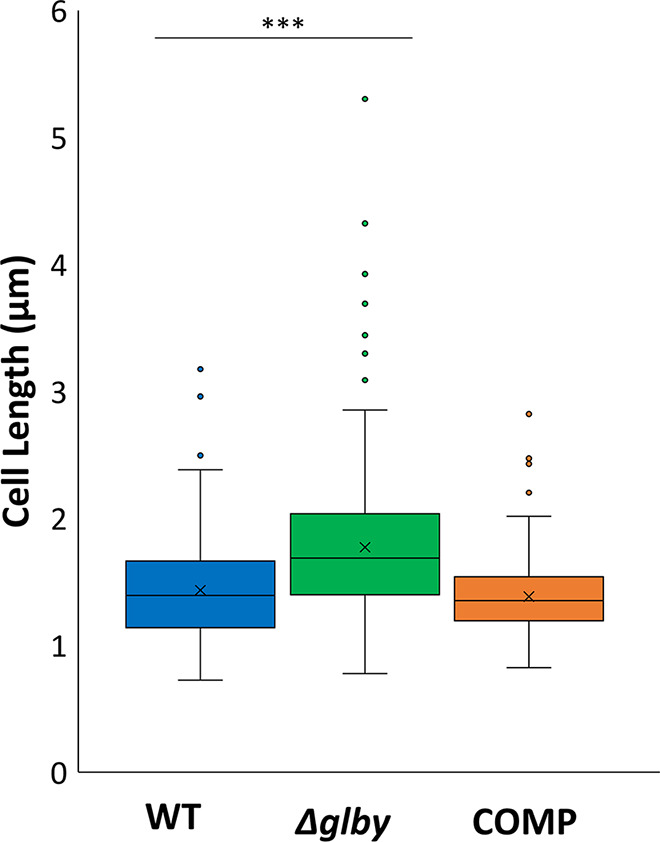
Box plots of cell lengths by strain. Box represents the interquartile range (middle 50% of the data). Vertical lines coming out of each box represent the minimum and maximum of the data. Dots represent outliers, horizontal line in the box represents the median cell length and “x” within the box represents the mean cell length of each strain. Cell lengths were determined by SEM and measured using ImageJ. Parent strain *Mab* ATCC 19977 (WT), *Δglby*, and Complemented strain (COMP).

Next, we used scanning electron microscopy to investigate the cellular appearances of WT, *Δglby*, and COMP cells. *Δglby* cells exhibited two distinct cell morphotypes. First, on average, *Δglby* cells were ∼300 nm longer than WT or COMP cells (*n* = 250 cells, *P*-value <0.0001) (Fig. 3, Table S2). There were also many outliers from each strain; however, the majority of *Δglby* cells were consistently longer than WT or COMP cells with some over 4 µm (Fig. S9).

**FIG 4:**
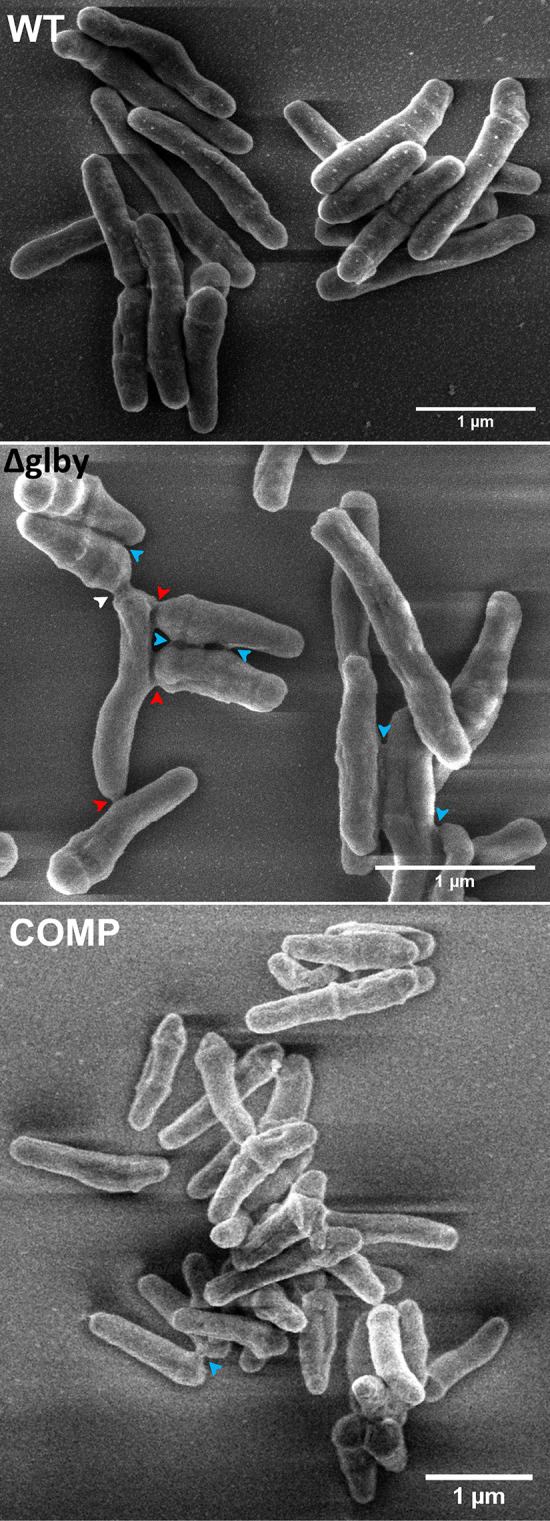
Scanning electron microscopy images of M. abscessus strains during exponential growth phase. Arrowheads indicate locations where melding of cell surfaces have occurred, including melding between two poles (white), between a pole of one cell and lateral surface of another (red), and between lateral surfaces of two cells (blue). Parent strain *Mab* ATCC 19977 (WT), *Δglby*, and Complemented strain (COMP).

The second notable difference is the melding of cell surfaces of *Δglby* cells which occurred at a higher rate than in WT and COMP cells and between more than two cells (Fig. 4). Surface melding occurred in 21.4% of *Δglby* cells, whereas these occurrences were observed in 4.1% and 3.5% of WT and COMP cells, respectively (Table S3). Also, melding among more than two cells occurred frequently in *Δglby*, with a maximum of 11 cells melded together in a single clump, whereas WT and COMP cells exhibited melding between two cells only. The melding is characterized by the presence of an extracellular matrix sandwiched between the surfaces of two cells and observable as mass of light density. Often, this matrix appeared to be making physical contact between the surfaces of two or more cells and stretching along the axis perpendicular to the length of contact surface. Melding was not restricted to a specific region of the cell, rather we observed melding between two poles, between a pole of one cell and lateral surface of another or between lateral surfaces of two cells. Interestingly, cell-cell melding was most frequently observed in shorter cells as opposed to the longer cells that did not show this multi-point, multi-cell melding in our data set.

### Glby exhibits mild β-lactamase activity.

**FIG 5:**
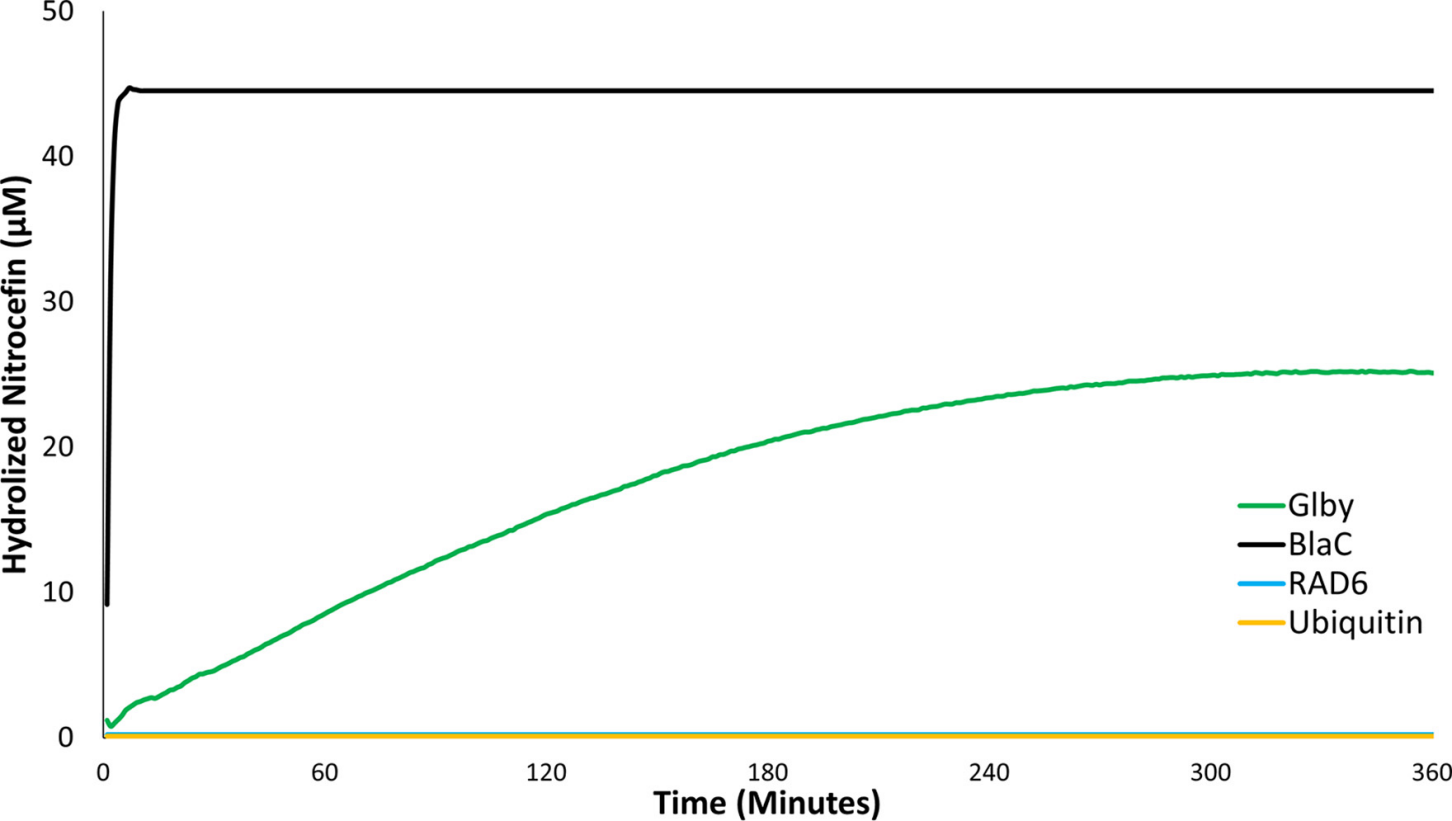
Activity of Glby against Nitrocefin. Time course of nitrocefin (100 µM) hydrolysis by BlaC, Glby, RAD6, and ubiquitin (1.7 µM, each protein) was measured at 490-nm wavelength over a period of 360 min. Each data point shown is the mean of triplicates. To account for the low level of hydrolysis of nitrocefin in the reaction buffer, the absorbance in the control reaction (nitrocefin only) at each time point was used to correct the absorbance readings in reactions containing protein. As RAD6 and ubiquitin failed to hydrolyze nitrocefin, their plots appear superimposed.

The *C-*terminus of M. tuberculosis protein Rv2864c harbors a predicted FtsI domain, the presence of which classifies it as a putative PBP (46). As Glby also possesses the same putative FtsI domain, we hypothesized that Glby may also bind to β-lactams as may be expected from proteins with an FtsI domain. This activity is unrelated to the native cellular function of Glby which is likely to be associated with biosynthesis or metabolism of the cell wall. To test this hypothesis, *glby* was overexpressed using pET28a+TEV specifically to avoid any β-lactamase activity arising from proteins expressed from the backbone of the plasmid, such as β-lactamases that are commonly used for selection when cloning. pET28a+TEV harbors a kanamycin resistance marker for selection and is devoid of any β-lactamase encoding gene. Glby was purified to homogeneity (Fig. S10), and we assessed its ability to bind and hydrolyze a chromogenic β-lactam, nitrocefin. This is one of many validated assays for assessing if a protein can bind and metabolize compounds belonging to the β-lactam class, which includes penicillins, cephalosporins, carbapenems, etc. (47, 48). We included two negative control proteins, RAD6 (Saccharomyces cerevisiae) and Ubiquitin (human) and as a positive control, we included a β-lactamase, BlaC, of M. tuberculosis that is known to rapidly hydrolyze β-lactams (49, 50). All proteins were individually incubated with 100 µM nitrocefin. BlaC reached a rate of nitrocefin hydrolysis of 22.9 µM/min whereas Glby hydrolyzed nitrocefin at a rate of only 0.14 µM/min(Fig. 5). As such, BlaC was able to reach maximum hydrolysis of nitrocefin within 2 min, whereas Glby was able to reach maximum hydrolysis in 5 h and only reached levels about half of BlaC. RAD6 and ubiquitin failed to hydrolyze nitrocefin.

### Glby is essential for *in vivo* viability and proliferation in mouse lungs.

Next, we asked if Glby is required for *Mab* to produce a productive infection and disease in a mouse model of pulmonary *Mab* infection. As lung infections are the most commonly reported condition in patients diagnosed with *Mab* disease (1, 8), a mammalian model of lung *Mab* infection would permit assessment of whether Glby is required for *Mab* to survive and proliferate in the lungs. It has been recently demonstrated in a mouse model of pulmonary *Mab* infection that the parent strain of *Δglby* (*Mab* strainATCC 19977) proliferates in the lungs, produces pathology similar to that in humans, and mimics the response to antibiotics used to treat *Mab* lung infections in humans (51, 52). We used this preclinical mouse model of pulmonary *Mab* infection to investigate whether *Δglby* can survive and proliferate in the lungs and included WT and COMP as comparator controls.

**FIG 6:**
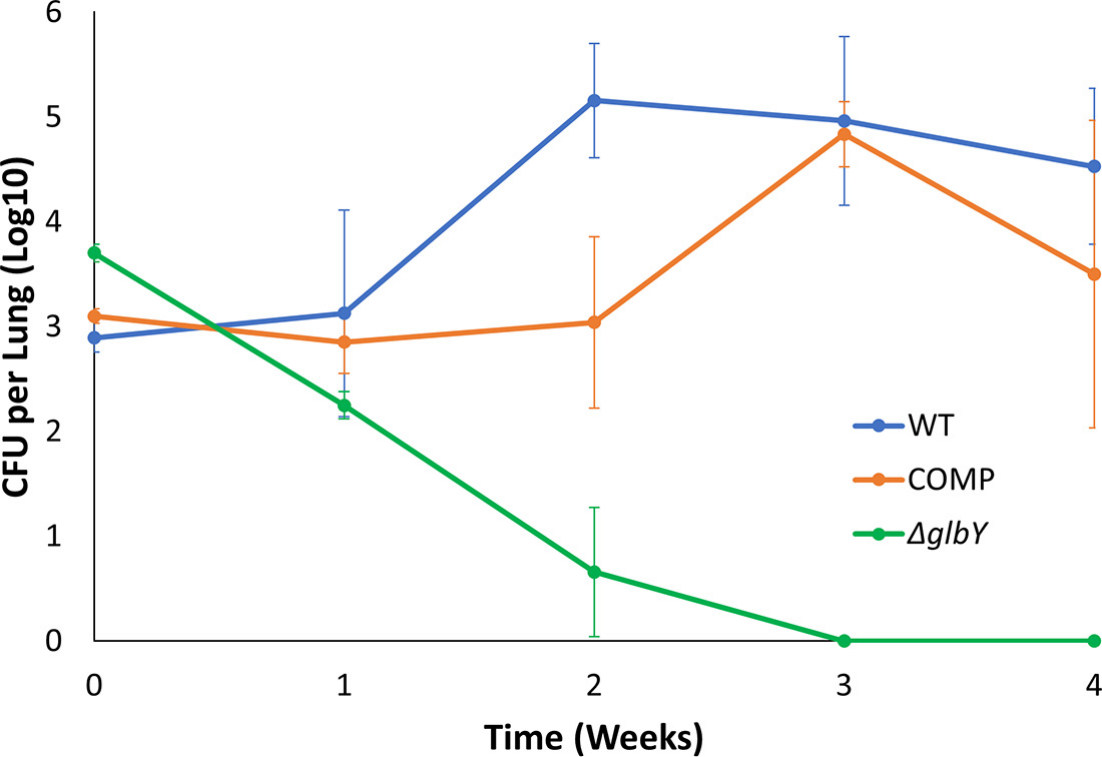
*Mab* burden in the lungs of mice. Burden of *Mab* strain ATCC 19977 (WT), *Δglby*, and Complement (COMP) in the lungs of C3HeB/FeJ mice (*n* = 5 per strain per time point).

As it is necessary to grow these strains *in vitro* to generate a suspension with which to infect mice, and *Δglby* growth in culture broth does not mimic its parent strain, we performed a pilot study to determine the optimal infection dose of *Δglby* required to match the implantation CFU of WT and COMP strains. Although *Δglby* was dispersed prior to infection, it implanted at 10x lower levels than WT and COMP strains in the lungs of mice (Fig. S11). Based on this finding, it was necessary to increase the inoculum of *Δglby* by 50x so that following its aerosolization, *Δglby* would implant in the lungs of mice at a burden no less than that of WT and COMP. At 24 h following infection, the lung burdens of *Δglby*, WT and COMP strains were 3.7 log_10_, 2.9 log_10_ and 3.1 log_10_, respectively. While the lung burdens of WT and COMP remained steady during the first week, by the third week there was ∼2 log_10_ increase in their CFU in the lungs of mice (Fig. 6, Table S4). However, the lung burden of *Δglby* steadily decreased from the day of implantation. By week 2, 99.6% of *Δglby* had been cleared from the lungs of mice; by contrast WT CFU burden in the lungs increased as previously demonstrated (51) and had a final increase in bacterial burden of 2 log_10_ over the implantation level. By the 3-week time point, *Δglby* CFU was undetectable on growth medium inoculated with the entire lung homogenates from all five mice to detect any surviving *Mab.* At the 4-week time point, we were again unable to detect any *Δglby* in the lungs of mice, while WT maintained its CFU level and COMP exhibited a slight decrease, but within the standard deviation of WT CFU levels. As *Δglby* not only failed to proliferate in the lungs of mice at any time during the study period, but instead was steadily cleared to undetectable levels, we conclude that Glby is required for *Mab* viability, growth and proliferation in the lungs of C3HeB/FeJ mice. In the study that originally reported this mouse model, the mice eventually succumbed to death from pathology resulting from the WT strain (51). As immunocompromised mice were able to clear *Δglby* from their lungs, it suggests that Glby is also required for virulence of *Mab* to cause lung disease.

## DISCUSSION

The significance of the peptidoglycan biosynthesis pathway to bacteria is underscored by its requirement for viability, growth, and division (26, 53). Considered the Achille’s Heel in bacterial cell physiology, agents that inhibit the peptidoglycan biosynthesis pathway, namely, β-lactams and glycopeptides, comprise more than half of all antibiotics prescribed to treat bacterial infections in humans (54). The final step of peptidoglycan synthesis in *Mab* is catalyzed by l,d- and D,D-transpeptidases (55). As the relevance of these enzymes in the cell physiology of *Mab* has not been reported, we limited the scope of the current study to D,D-transpeptidases and initiated a screen to identify a putative D,D-transpeptidase to begin generating insight into this important protein class in *Mab.* Using amino acid sequences of D,D-transpeptidases of M. tuberculosis, a search for sequence homologs in *Mab* yielded *MAB_0035c*, *MAB_0408c*, *MAB_2000*, *MAB_3167c*, and *MAB_4901c.* Using a validated approach for generating *Mab* strains with gene deletions (40), we were unable to generate *Mab* with deletion of *MAB_0035c* or *MAB_2000*, despite repeated attempts. *MAB_2000* is a homologue of *Rv2163c*, an essential gene in M. tuberculosis (56, 57), potentially indicating that it may also be essential in *Mab*. A recent transposon mutagenesis screen was also unable to isolate a *Mab* mutant with this gene disrupted (58). This screen also reported that loss of *MAB_0035c* affects growth of *Mab*. Growth of *Mab* lacking *MAB_0408c* and *MAB_4901c* in Middlebrook 7H9 broth and Middlebrook 7H10 agar were unremarkable compared with the parent strain and therefore were not considered further in this study.

The relevance of peptidoglycan synthesis transpeptidases have been described in organisms related to *Mab* (30). In M. tuberculosis and M. smegmatis, specific D,D-transpeptidases affect cell morphology (31) and are required for *in vitro* growth (28, 56, 59). Our observations of the aplanktonic and globular, *in vitro* growth of *Δglby*, in addition to the aggregated growth phenotype via cell surface melding with lightly staining extracellular matrix, demonstrate its relevance to morphology during growth. It is a known phenomenon that several non-tuberculous mycobacteria tend to aggregate when grown in standard laboratory growth media (60). The *Δglby* growth phenotype is distinct, as it forms clumps that are much larger (∼0.65 cm) and not comparable with those described in the literature. The presence of extracellular matrix consisting of DNA, proteins and mycolic acids in *Mab* grown in standard laboratory growth media, as well as extracellular matrix comprised of DNA, glycan, and phospholipids, when grown in synthetic media representing lung secretions in cystic fibrosis patients, have been reported (61–63). As this is the first characterization of *Mab* lacking Glby, it was beyond the scope of the current study to characterize the composition of the extracellular matrix at the sites of surface meldings between *Δglby* cells. Therefore, we are unable to speculate whether the composition of the extra cellular matrix is similar to or different from what has been described in the literature. We did not observe prominent extracellular matrix in WT and COMP cells. In a screen to probe the source of extracellular DNA, in Mycobacterium avium, reduced levels of extracellular DNA was observed in a mutant with transposon insertion disrupting gene *MAVA_03380* which encodes a putative FtsK/SpoIIE (61). FtsK is known to be associated with cell division, whereas FtsI, a domain present in Glby, is known to catalyze peptidoglycan synthesis for cell elongation. It is possible that the loss of Glby function contributes to the increased average cell length of *Δglby*. Also, the possibility for β-lactam binding activity of Glby could be expected from the presence of this FtsI domain, which is known to exhibit β-lactam binding activity (64), as well as from homology of its protein sequence to that of a (Rv2864c, which is annotated as a PBP-lipo) (30, 32, 43). Based on the observation that *Δglby* cells exhibit distinct growth and cell surface morphologies, and the presence of an FtsI-like domain, we hypothesize that Glby may be associated with cell wall function. Although significantly milder than BlaC in β-lactamase activity, Glby was able to hydrolyze a β-lactam probe (Fig. 5). This could not be anticipated from prior literature as proteins containing an FtsI domain are considered to be putative PBPs and therefore a target for inactivation by β-lactams (26, 46).

Our findings also demonstrate that Glby is dispensable for growth *in vitro*, but its loss significantly increases the generation time of *Mab.* A recent study based on the generation of a pool of mutants with transposon insertion and detection of outgrowth mutants concluded that *glby* is likely essential for growth *in vitro* (58). In this approach, thousands of mutants were generated simultaneously, recovered after outgrowth in a pool, and transposon insertion sites represented in the surviving mutants were identified. The genes disrupted in the mutants in the pool are predicted to be not essential for *in vitro* growth. This approach is a powerful first screen for prediction of essential genes and has high, but not absolute, accuracy of identifying essential genes (56, 57). Essentiality prediction is based on the lack of detection of a mutant in the pool, which can also include growth impaired mutants, as thousands of other mutants in the pool outgrow impaired mutants and are therefore more readily detected in the output. Additionally, the possibility that a mutant’s viability or growth is significantly affected in the presence of a complex mutant pool cannot be ruled out. We also demonstrate that specific agitation is required to support growth of *Δglby* and this could be a potential reason why this strain was not detected in the mutagenesis study. To ascertain if a predicted gene is essential, a direct and site-specific mutagenesis is necessary. Therefore, detection of mutations that attenuate *in vitro* growth, such as *Δglby*, is a challenge with transposon based random mutagenesis due to limitations of detection in outgrowth pools. Our findings demonstrate that while Glby is dispensable for *in vitro* growth, it is required for viability and growth in the lungs of mice. Based on this finding, we hypothesize that Glby is required for *Mab* to establish a productive infection and cause disease in humans. Despite declumping *Δglby* cells prior to infecting mice, it is possible that their implantation in the lungs was different from WT and COMP due to potential changes in the cell wall properties. To determine whether the ability of *Δglby* to form clumps affects their rate of proliferation in the lungs of mice and subsequent disease progression will require additional studies. Based on the clumping and attenuated *in vitro* growth phenotypes, we hypothesize that cell wall composition of *Δglby* is altered and these alterations underlie their loss in viability in the lungs of mice.

The 1,046 *Mab* clinical isolates retrieved from PATRIC represent genotypes with fitness to cause disease in humans. Therefore, Glby sequences in these mutants represent types that likely did not compromise viability or virulence of *Mab* in humans. In the 1,046 *Mab* clinical isolates analyzed, none of the SNPs resulted in nonsense mutations that would lead to a truncated Glby, and only one missense mutation with low predicted protein impact. As we were unable to identify any deleterious mutations in Glby in clinical isolates, these data suggest that Glby is likely required for *Mab* viability in humans as well. One approach toward developing a new therapeutic agent is identification of a protein, or cellular target, in the pathogen that is required for viability or virulence in the host, so that, when inhibited, it can arrest the disease and eventually lead to curing it. Using genetic and microbiological approaches and a mouse model of pulmonary *Mab* infection, we have provided evidence that biochemical inactivation of pathogenic *Mab* with an antibiotic specific to Glby has the potential to exhibit bactericidal activity against *Mab* and improve treatment outcomes.

## MATERIALS AND METHODS

### Bacterial strains and growth conditions.

M. abscessus strain ATCC 19977 (39) was procured from ATCC (Manassas, Virginia), and used as the parent strain, and all strains were derived from it. In general, validated protocols for preparing common reagents for handling and growing mycobacteria were used (65). All strains were grown in Middlebrook 7H9 broth (Difco) supplemented with 10% albumin-dextrose-saline enrichment (ADS), 0.5% glycerol, and 0.05% Tween 80 with constant shaking at 200 rpm in an orbital shaker at 37°C. To culture on solid media, Middlebrook 7H10 agar (Difco) supplemented with 10% ADS and 0.5% glycerol and appropriate antibiotic, depending on the selection required (as noted below), were used. *Mab* ATCC 19977 exhibited both smooth and rough colony morphotypes on Middlebrook 7H10 agar. The vast majority of the colonies appeared smooth. All inoculations that required agar base were performed on Middlebrook 7H10 with the exception of mouse lung homogenates, which were inoculated onto selective Middlebrook 7H11 agar (Difco) supplemented with 10% ADS, 0.5% glycerol, 20 mg/L Trimethoprim (Sigma-Aldrich, T7883), 50 mg/L Carbenicillin (Fisher Scientific, 50-213-247), and 50 mg/L Cycloheximide (Sigma-Aldrich, C7698). E. coli strain DH5α (NEB Labs, C2987H) was used for cloning and E. coli strain BL21(DE3) (NEB Labs, C2527) was used for protein overexpression. These strains were grown in LB Broth as specified by the manufacturer.

### Genetic manipulation of *Mab*.

For preparation of electrocompetent cells, *Mab* strain ATCC 19977 was grown in Middlebrook 7H9 broth to mid-log phase. A subculture in 100 mL of Middlebrook 7H9 supplemented with 0.2% succinate was initiated and monitored until it reached optical density (measured as absorbance at 600 nm, A_600nm_) of 0.5 to 0.8 at which point the culture was placed on ice for 30 min and divided into two 50 mL aliquots. These cells were washed four times as follows: the cell suspension was centrifuged at 3,000 rpm for 10 min at 4°C, supernatant was discarded, and the pellet was resuspended in 40 mL ice-cold 10% glycerol. After the final wash, the cell pellet was resuspended in 1 mL 10% glycerol and 200 µL aliquots were transferred into microfuge tubes and stored at −80°C until use.

For genetic manipulation of *Mab*, electrocompetent ATCC 19977 was transformed with pJV53 in accordance with the recombineering protocol (40). Briefly, *Mab* competent cells were incubated on ice with ∼500 ng of DNA for 10 min and transformation was performed (2.5 kV, 25 uF, 1000 Ω) using an electroporator (Bio-Rad). Cells were recovered in 1 mL of Middlebrook 7H9 broth, incubated for 4 h at 37°C and inoculated onto Middlebrook 7H10 agar supplemented with appropriate antibiotics. For selection of pJV53, 128 µg/mL kanamycin was used, for allelic exchange substrate 64 µg/mL Zeocin and for pMH94-Apra, 25 µg/mL Apramycin was used.

### Cloning.

All PCRs were carried out using Phusion High Fidelity Polymerase (NEB Labs, M0530). To begin, we PCR amplified *Zeo^R^* cassette flanked by *loxP* sites using pMSG360zeo as template (66) and inserted this fragment into pUC19 at the multiple cloning site. Next, we PCR amplified the ∼1,500 bp regions from 5′ and 3′ ends of *glby* using PCR and cloned into the flanks of *Zeo^R^* cassette. This plasmid was used as the template to generate linear allelic exchange substrate spanning 5’*glby*-*loxP-Zeo^R^*-*loxP-3’glby*. pMH94-Apra was generated by replacing Kan^R^ cassette from pMH94 (41) with Apra^R^ gene cloned from pCAP03-acc(3)IV (67) using primers that had a complementing overhang with the PCR product from pMH94 (41) for easy restriction cloning.

For overexpression of Glby, *glby* was PCR amplified using genomic DNA of *Mab* strain ATCC 19977 as template and cloned into pET28a+TEV (68). The region in the *N-*terminus of Glby that is predicted to encode a transmembrane anchor domain based on TMHMM (69) and TMPred (70), amino acid residues 1 to 19, was excluded during cloning to facilitate extraction and solubilization of Glby. DNA sequences of all resulting plasmids were determined via Sanger sequencing (Eurofins, KY) and only clones with correct sequences were selected for study.

### Protein overexpression and purification.

Glby was overexpressed in E. coli BL21(DE3) cells carrying pET28a-*glby* by inducing a 1- L culture in LB broth with 0.25 mM IPTG overnight at 16°C with orbital shaking at 150 RPM. Without the addition of solubility enhancing additives, Glby would exclusively be present in the pellet fraction. To address this, induced cells were resuspended in sonication buffer (Tris-Cl 50 mM, NaCl 300 mM, and Imidazole 25 mM, pH 8) with sarkosyl (1% final concentration) and cOmplete EDTA-Free protease inhibitor cocktail (Millipore-Sigma, 11836170001). Cells were then sonicated for 6 min (intervals of 15 s on, 30 s off) on ice. Solubilized Glby was purified using the His-Tag via Ni-NTA based metal affinity chromatography using an AKTA FPLC (GE Healthcare, USA). The protein sample was then washed with at least 5 column volumes in washing buffer (identical to sonication buffer except for sarkosyl). Glby was eluted with Tris-Cl 50 mM, NaCl 300 mM, and imidazole 500 mM, pH 8 (Fig. S10) and further purified to remove Imidazole and other impurities via size exclusion chromatography (HiPrep 26/60 column). From this preparation, we attempted to concentrate Glby using protein concentrators (Vivaspin 2 MWCO 30,000, Cytiva, 28932248), however, it became evident that Glby would adhere to the membrane and could not be concentrated to large amounts. Glby concentration was determined using a spectrophotometer to a concentration of 1.7 µM. M. tuberculosis BlaC protein prepared in a previously study (71) was used. RAD6 (S. cerevisiae) and Ubiquitin (human) were purified using a protocol similar to that described here. These proteins were diluted in the same buffer used for Glby and BlaC.

### gDNA extraction, whole genome sequencing, and assembly.

Genomic DNA (gDNA) from all strains was extracted and purified as described for mycobacteria (65). The purity of gDNA preparations were determined spectrophotometrically. Sequences of genomes of WT *Mab* strain ATCC 19977, Δ*glby*, and COMP were determined using Illumina PE150 platform (Novogene, CA, USA). We re-sequenced our stock of WT *Mab* ATCC 19977 strain to identify pre-existing SNPs in comparison with the reference sequence of ATCC 19977. The gDNA sequence of our stock of ATCC 19977 was used for comparison with the sequences obtained for Δ*glby* and COMP. To verify genotype of *Δglby*, Geneious v11.1.5 (Biomatters) was used to map the sequence reads obtained from gDNA of this strain to the reference strain (Fig. S3). To verify the genotype of the complement strain, *de novo* assembly of the reads was performed, and genes were subsequently annotated in the generated contigs with 100% identity to genes in *Mab* ATCC 19977. Neighboring genes to the *attB* were used to localize the integrated plasmid (Fig. S5).

### Distribution of mutations in Glby in *Mab* clinical isolates.

Whole genome sequences of 1,046 *Mab* clinical isolates from around the world that are archived in the PATRIC database (38), including their locations of origin, were considered in our study. Using Geneious v11.1.5 (Biomatters), the sequence of *glby* from all genomes was extracted by creating a custom BLAST database within Geneious. Using ATCC 19977 *glby* as the reference sequence, all 1,046 genomes were queried, and the identified sequences were aligned with the MUSCLE alignment tool included in the software, and a consensus sequence of *glby* was generated (Fig. S1). SNPs and variations in *glby* in each of the 1,046 strains were identified by comparison against the consensus sequence using the “Find Variations/SNPs” algorithm in Geneious. The same method was applied for the mutation distribution of locus MAB_3157c to MAB_3177 and a one-sample *t* test was used to determine the *P*-value for *glby* compared to its surrounding genes.

### Determination of *Mab in vitro* growth profiles.

Stocks of *Mab* WT, Δ*glby*, and COMP strains, archived in −80°C, were used to inoculate Middlebrook 7H9 broth to generate primary cultures. These cultures were used to inoculate 120 mL Middlebrook 7H9 broth with the same starting OD of A_600nm_ = 0.0005. This suspension was used to transfer 2 mL aliquots into 14 mL culture tubes. Four distinct tubes/samples were allocated per time point for each strain and OD was determined by measuring A_600nm_. Simultaneously, for each strain, 100 μL of appropriate dilutions of each sample at planned time points were inoculated onto Middlebrook 7H10 medium, incubated at 37°C for 5 days for WT and COMP and 8 days for Δ*glby* and CFU were enumerated. Δ*glby* CFU required additional 3 days to appear compared to WT and COMP strains and thus the difference of incubation duration.

### Ziehl-Neelsen and Auramine-rhodamine staining.

For both ZN and AR staining stains (44, 45), 60 μL of culture from each strain at exponential phase were placed on a glass slide and allowed to air dry completely. The slides were then passed over the flame of a Bunsen burner for 5 s to heat fix the sample. For ZN stain (Kit–90008-884, BD), carbolfuchsin was applied to cover the smear, heated for 5 s using a Bunsen burner and letting it rest for 5 min. After rinsing the sample with deionized (DI) water, the decolorizer was applied, incubated for 1 min and rinsed again with DI water. The counter stain methylene blue was added to the sample, incubated for 30 s and rinsed with DI water. Once the slides were completely dry, a glass cover slide using Permount mounting medium (SP15-100, Fisher Scientific) was applied. For the AR stain (Kit–212521, BD), the smear was covered with Auramine-rhodamine T for 15 min, after which it was rinsed with DI water. The Decolorizer TM was applied, incubated for 2 min and rinsed with DI water. Finally, potassium permanganate was applied, incubated for 3 min and rinsed with DI water. Once the slides were completely dried, a glass cover slide using Permount mounting medium was applied.

### Nitrocefin hydrolysis assay.

Glby, BlaC of M. tuberculosis (49, 50), RAD6 (S. cerevisiae), and ubiquitin (human) each at 1.7 µM, were individually mixed with nitrocefin (Calbiochem) 100 µM, in 50 mM Tris-Cl buffer pH 8 in a final reaction volume of 100 μL and incubated at 25°C for 6 h. As a control, nitrocefin in buffer, but without any protein was included. Hydrolysis of nitrocefin was monitored by measuring the absorbance specific to the hydrolyzed product (λ_max_ = 490 nm) (47, 48). To account for the low level of hydrolysis of nitrocefin in the buffer itself, the absorbance in the control reaction at each time point was used to correct the absorbance readings in reactions containing protein. Each assay was performed in triplicates.

### Scanning electron microscopy and cell length determination.

Stocks of *Mab* WT, Δ*glby*, and COMP strains, archived in −80°C, were used to inoculate Middlebrook 7H9 broth to generate 3 mL cultures. From each culture, 1.5 mL was taken during exponential phase, and each were fixed in 2.5% glutaraldehyde, 3 mM MgCl_2_, in 0.05 M sodium cacodylate buffer, pH 7.2 overnight at 4°C. The sample preparation was rinsed with DH_2_O, and subsequently postfixed in 1% osmium tetroxide in 0.075 M sodium cacodylate buffer for 1 h on ice in the dark. Following another DH_2_O rinse, samples were dehydrated in a graded series of ethanol and left to dry overnight in a desiccator with hexamethyldisilazane (HMDS). Samples were then mounted on carbon coated stubs and imaged on a Thermo Fisher Helios Focused Ion Beam Scanning Electron Microscope (FIB-SEM).

To measure cell length, we used ImageJ to first set the scale of the image by using the scale bar generated by the microscope software. We used the Freehand line tool to sketch a line between the apex of two poles and parallel to the lateral sides of those cells that were fully and clearly visible and measurements were recorded. Statistical analysis (*t*-tests) was performed using Microsoft Excel’s data analysis package and a box-and-whisker plot was generated. We manually counted only cells that were clearly visible in our data set and enumerated the melding events to generate percentage of melding events.

### *In vivo* viability and growth assessment of *Mab*.

A mouse model of pulmonary *Mab* infection and the protocol described (51) was used to assess the requirement of Glby for viability and growth of *Mab.* Briefly, C3HeB/FeJ mice, female, 5 to 6 weeks old (Jackson Laboratories, Bar Harbor, Maine), 25 mice per infecting strain, were infected with an aerosol of cultures of WT, Δ*glby*, or COMP obtained at exponential phase of growth and diluted to an OD of A_600nm_ = 0.01 for WT and COMP, but 0.5 for *Δglby*, in sterile 1x PBS, pH 7.4 in a Glas-Col nebulizer according to the manufacturer’s instructions (Glas-Col, Terre Haute, Indiana). Because *Δglby* cells clump, we dispersed the clumps by repeated shaking and pipetting through 1 mm orifice until a homogeneous suspension was obtained. One week prior to infection and throughout the study, mice were treated daily with a single dose of dexamethasone, 5 mg/kg/day as specified in the mouse model protocol. Five mice per infection group were sacrificed at 1 day (week 0), 1, 2, 3, and 4 weeks following infection, lungs were homogenized, inoculated onto Middlebrook 7H11 selective agar, incubated at 37°C for 5 days for WT and COMP and 8 days for Δ*glby*, and CFU were enumerated. Mean CFU ± standard deviation was calculated to determine the CFU burden of each strain over the time course of the study. Statistical analyses (*t*-tests) were performed using Microsoft Excel’s data analysis package for each time point for comparisons between infection groups (Table S4).

Animal procedures used in the following studies were performed in adherence to the Johns Hopkins University Animal Care and Use Committee and to national guidelines.

### Data availability.

The genomics data for clinical strains of M. abscessus are available in PATRIC at https://www.patricbrc.org/. The genomics data for strains created in this study are available from the corresponding author upon reasonable request.
