# Supplementary material for: Glby, Encoded by MAB_3167c, Is Required for In Vivo Growth of Mycobacteroides abscessus and Exhibits Mild β-Lactamase Activity
Source: J Bacteriol. 2022 Apr 5;204(5):e00046-22. doi: 10.1128/jb.00046-22 (PMC9112878; doi:10.1128/jb.00046-22)
Supplement: Supplemental file 1 — Tables S1 to S4, Fig. S1 to S4. Download jb.00046-22-s0001.pdf, PDF file, 2.8 MB [file jb.00046-22-s0001.pdf]

SUPPLEMENTARY INFORMATION

SUPPLEMENTARY TABLES

Table S1. Frequency of missense mutations in genes in the *glby* (*MAB_3167c*) locus

Gene ID	# of missense mutation above 5% frequency	Gene Length (kbp)	Mutations/gene length
MAB_3157c	12	1.869	6.421
MAB_3158c	2	0.447	4.474
MAB_3159c	3	1.431	2.096
MAB_3160	5	0.984	5.081
MAB_3161c	1	0.627	1.595
MAB_3162c	5	0.63	7.937
MAB_3163c	11	1.524	7.218
MAB_3164c	2	0.804	2.488
MAB_3165c	1	0.747	1.339
MAB_3166	7	0.375	18.667
**MAB_3167c**	**1**	**1.818**	**0.550**
MAB_3168c	6	0.813	7.380
MAB_3169c	4	1.188	3.367
MAB_3170c	6	1.248	4.808
MAB_3171c	4	1.17	3.419
MAB_3172c	15	0.753	19.920
MAB_3173c	7	0.471	14.862
MAB_3174	4	0.339	11.799
MAB_3175c	3	1.122	2.674
MAB_3176c	1	0.597	1.675
MAB_3177	15	0.939	15.974

Table S2. Cell length statistics and t-test vs WT

	**Δglby**	**WT**	**COMP**
**Mean (μm)**	1.771	1.433	1.382
**Std Dev**	0.5240	0.3898	0.2952
**Observations**	251	251	250
**p-value (vs WT)**	<0.0001	-	>0.10
**95% CI (μm)**	1.714 - 1.793	1.385 - 1.481	1.346 - 1.419

Table S3. Cell melding statistics.

	WT	Δ*glby*	COMP
***Frequency of observed melding events**	4.1%	21.4%	3.5%
****Range of observed cells in melding clump**	2	2 - 11	2
**Total # of cells counted (n)**	314	611	578

*'Melding event' is defined as a clear and visible melding between two cells. **'Range of cells in melding clump' refers to the number of cells that are clearly melded together in a single clump, such as is shown in Figure 4, where there are more than two cells melded together for Δ*glby*.

Table S4. p-values for CFU lung burdens in each group at each time-point

Timepoint	p-value (WT vs Δ*glby*)	p-value (WT vs COMP)
**D0**	0.0005*	0.0128
**W1**	0.354	0.6654
**W2**	<0.0001*	0.001*
**W3**	<0.0001*	0.378
**W4**	<0.0001*	0.142

Asterisk (*) denotes a p-value of less than 0.01.

SUPPLEMENTARY FIGURES

ATGTTCAGACGTGGATGGGTCCTGGCGATGGTCGGCACCGTGCTGCTGGTGGCCGGTGCTGTCGCGTGTACCC
CGCGCCCCGATGGGCCGGGTCCGGTGGCGGAGAAGTTTTTCGAGGCACTGGCCAAGGGCGATACTGCCGCGG
CCGCTAAGCTAACCGACGACCCCGATGGCGCGAAAGTGGGCCTGGACCAAGCGTTCTCGGGACTACAGGCGAC
GTCGTTCAAGGCCGCGGTGAATGGCTCGCAGTACACCCAGGACACCGGTAGTGCGGATGCGACCTATACCTGG
CAGCTGCCCAGAAAGCGTGTCTGGACGTATAACGGGCGTCTGGAGATGTTGCGTACGGCAGGTAGCTGGCAG
GTACGTTGGGCTCCGAGTGACCTGCATCCCAAACTTGGTGAGCGCCAAATGCTCTCACTGCGCACAGACCCCGC
TAAGCGGGCGACGGTCAACGAAGCGGGCGGTACGACGGTGCTGGCCCCGGCCAATCTGTACAGAATCGCCTTC
GATGCCTCGAAGGCCGGCAAATCGTTGATGAGCACCGCCACCGCACTTGCCGACGCCATTCGGCCCTACGACG
ACACCATGAACGCCGCATCCCTGGCAGAGCAGGCGAGTGCGCAAACCTCGCCGATGGACCTGATCACGCTGCG
TAAGGATGACTGGGACAAGGTATCCATCGCGCTGGAGACGCGGCCGGGGGCGCTGCGGCCCGGCGTGGTGAT
GACGCCCATCGCCGATCTGTTGCCCACGGACGACGCTTTCGCGCCGGATATTGTCGCCCAGGTCAAAAAGGCG
GTGCTCGACGAGCTGGATGGCGAGGCCGGCTGGCGCGTGGTGAGCGTCAACCAGAACGGTGTCGACACTGCG
GTGCTCAACGAGGTGAAACCCACTCCGGCGCCGTCGAAGACGATCAGCCTTGATCGCGCCGTGCAGAACGCCG
CGCAGAATGCCGTCAATACCCGTGGCCAGAAGGCCATGATGGTAGTCATCAAACCGTCGACGGGTGAGATCCT
CGCGGTCGCCCAGAACGCCGCCGCCAACGCTGATGGACCGCTGGCCACCACCGGGTTGTATCCGCCTGGGTCG
ACATTCAAGATCATCACCGCCGGTGCCGCGCTGGAGCGCGGCATGGCCACTCCGGACACCATGGTGGGCTGCC
CGAAGCGGATCACCATCGGTGATCGCAGCGTGCCCAACTACAACGAGTTCGATCTCGGCACGGTGCCCATGTG
GCGGGCATTCGCGAACTCGTGCAACACCACCTTCGCGAAGCTGGCCAGCGAGATGCCTCTGGACGGTTTGACC
GTCGCCGCTTCACAATTCGGTATCGGACCCGACTACGACGTCGCGGGTATCCCGACCATTTCGGGCAACGTACC
ACCGACCGTCAATCTCACCGAGCGCACCGAGGACGGATTCGGTCAGGGCAAGGTGCTGGTCACGCCGTTCGGC
ATGGCGCTGGCAGCGGCCACCGTGGCAAATGGTAAAACACCTGTACCGCAGCTGATTTCGGGTCAGATCACCG
GAATAACCGGTGAGCGGCCCGCGGTGACCCCGACCATGGTCGATGGCCTGCGCGGAATGATGCGCGAGACGG
TGCTCAGCGGCACCGCCATGGATCTCAAGGGTGAGGGTGCGGTCTTCGGAAAAACGGGCGAAGCGGAGTTTC
CGGGCGGATCACATGCCTGGTTCGCCGGATACCGCGGCGACATGGCCTTCGCCACCTTGATCGTCGGGGGCGG
CGGTTCGGAGGCCGCGGTGCGCGCCACCAAGGTGATGTTCCAGTCGCTGCCGCCCGACTACTTGGCCTGA

Figure S1. Consensus sequence of *glby* (*MAB_3167c*). This consensus sequence of *glby* was generated using *glby* sequences in 1,046 independent clinical isolates from PATRIC database. Using Geneious® 1,046 sequences of *glby* were aligned and the most common base at each location was identified to generate the consensus sequence.

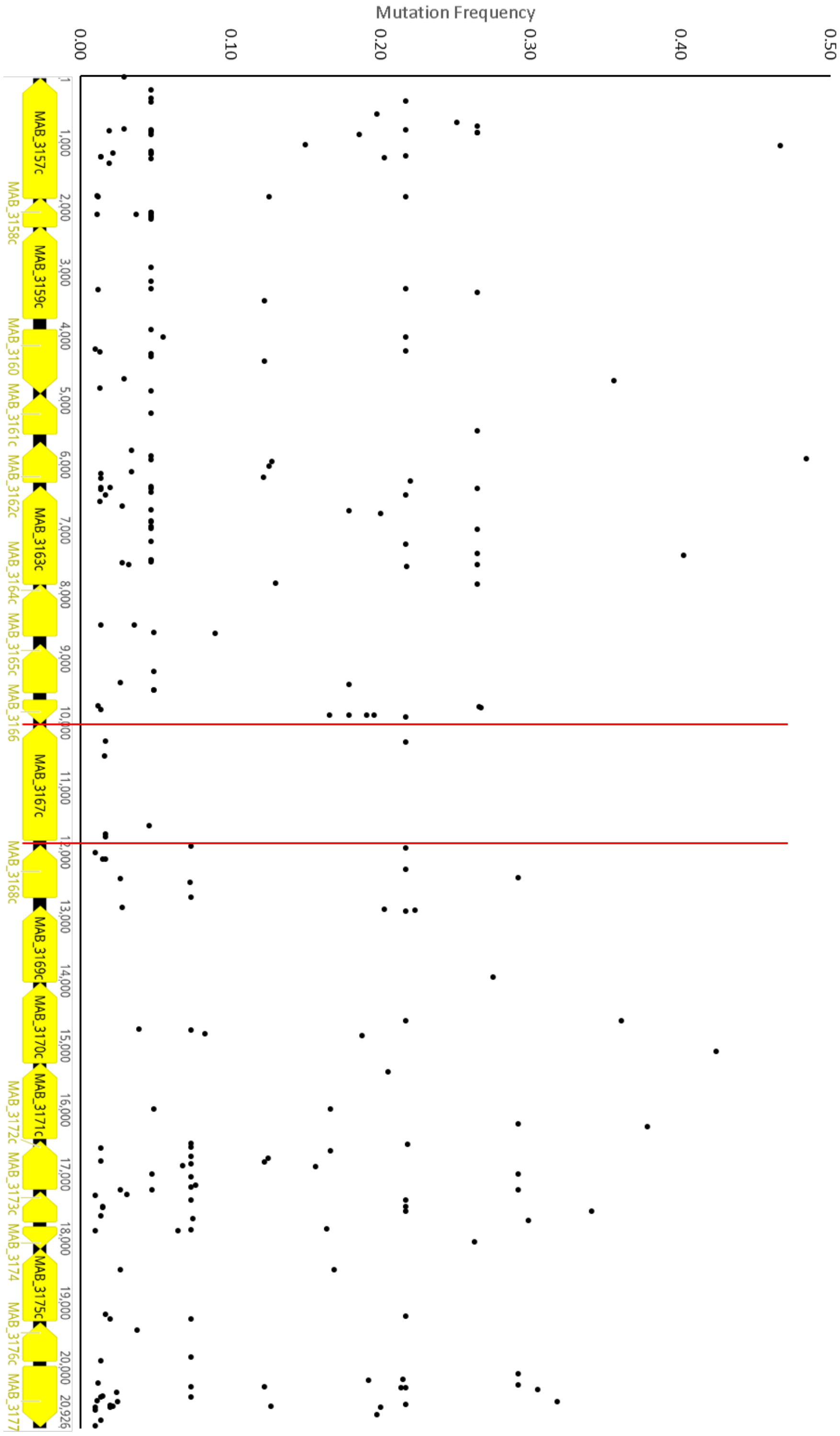

Figure S2. Frequency of mutations at the locus between genes *MAB_3166* and *MAB_3177*. Black dots represent substitutions in the amino acid sequence of each gene listed on the x-axis in yellow. Red lines highlight the genomic 5’ and 3’ ends corresponding to MAB_3167c (glby) for comparison to all other genes in this region.

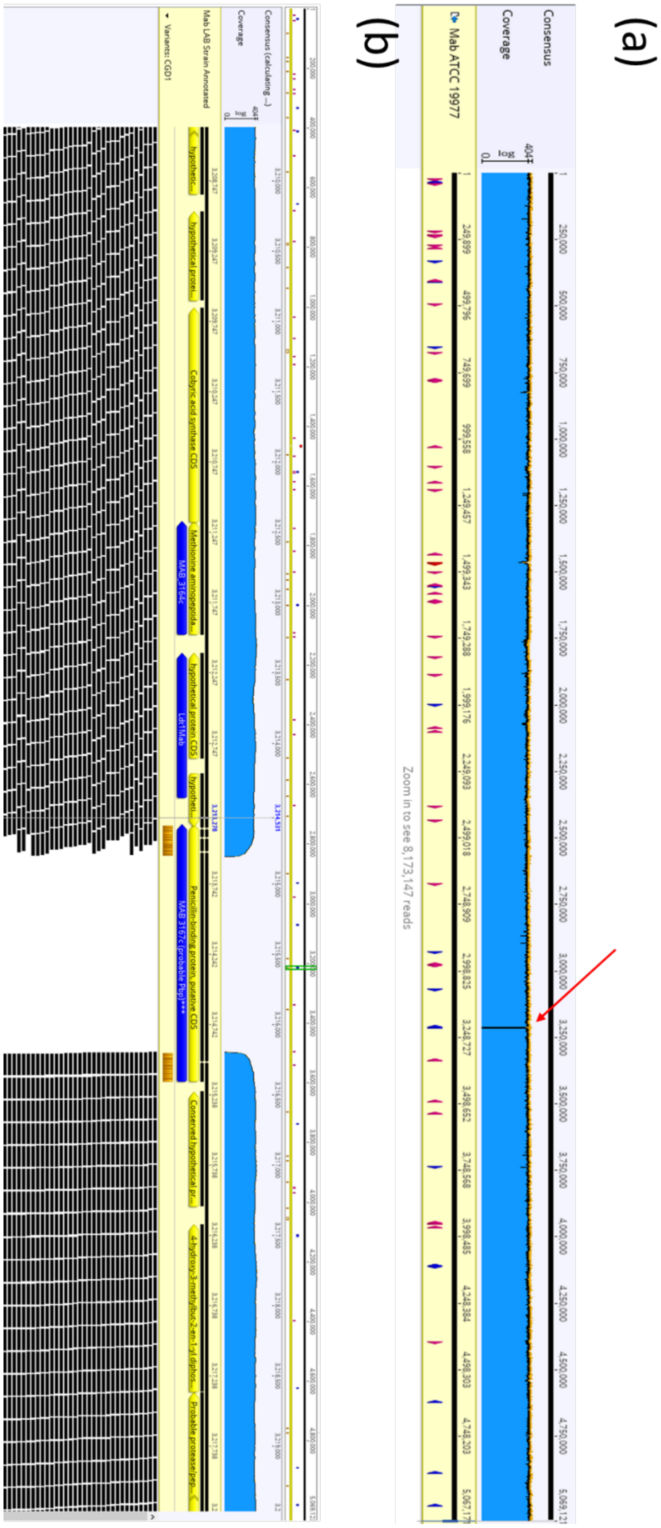

Figure S3. Mapping and alignment of whole-genome sequencing reads of Δ*glby* to the genome of *M. abscessus* reference strain ATCC 19977 (wild-type). (a) Coverage of ~8 million Illumina WGS reads on *M. abscessus* ATCC 19977 genome. Regions shaded blue indicate coverage of reads. (b) Close-up of region indicated by arrow in panel A.

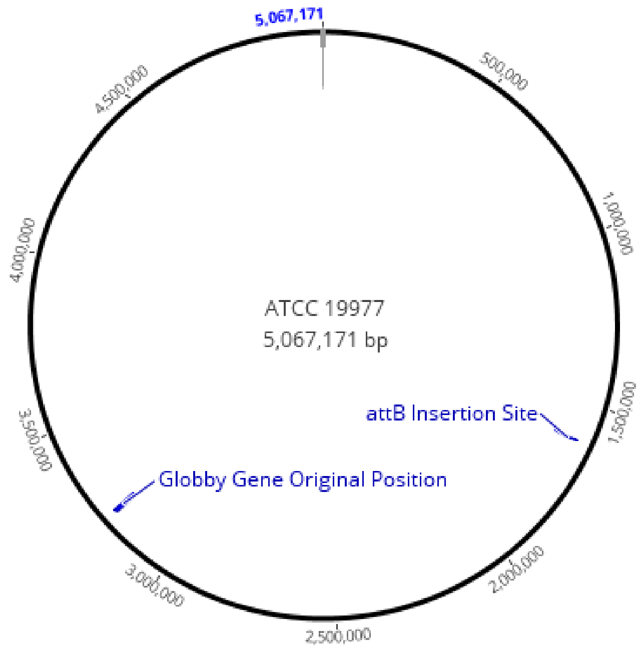

Figure S4. Genomic Insertion Site for pMH94 based plasmids. Map of *Mab* ATCC 19977 genome showing *glby*'s original locus within the genome and location of the *attB* insertion site where *glby* was inserted back into *Δglby* to generate the COMP strain.

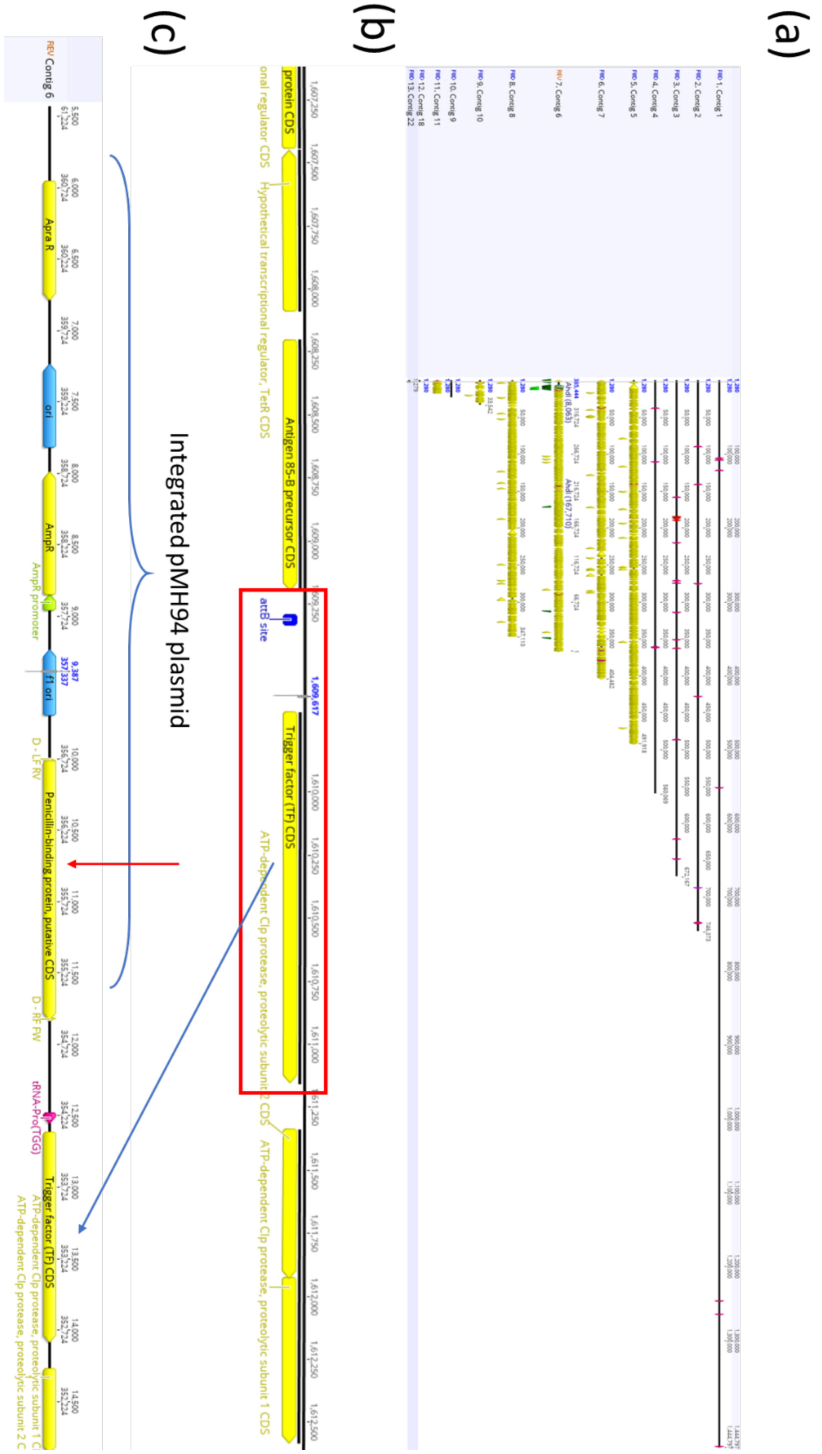

Figure S5. *De novo* assembly of COMP strain. (a) *de novo* assembly results of COMP strain which has been fully annotated based on 100% identity to original genes in *Mab* ATCC 19977. (b) Close-up of *attB* insertion site in *Mab* ATCC 19977. Red box indicates location where pMH94 based plasmid is expected to insert and Trigger Factor (TF) CDS is used as reference. (c) Location where pMH94Apra-MAB_3167c plasmid integrated into the genome. Blue arrow indicates reference gene for localization. Red arrow indicates *glby* is found at the *attB* insertion site.

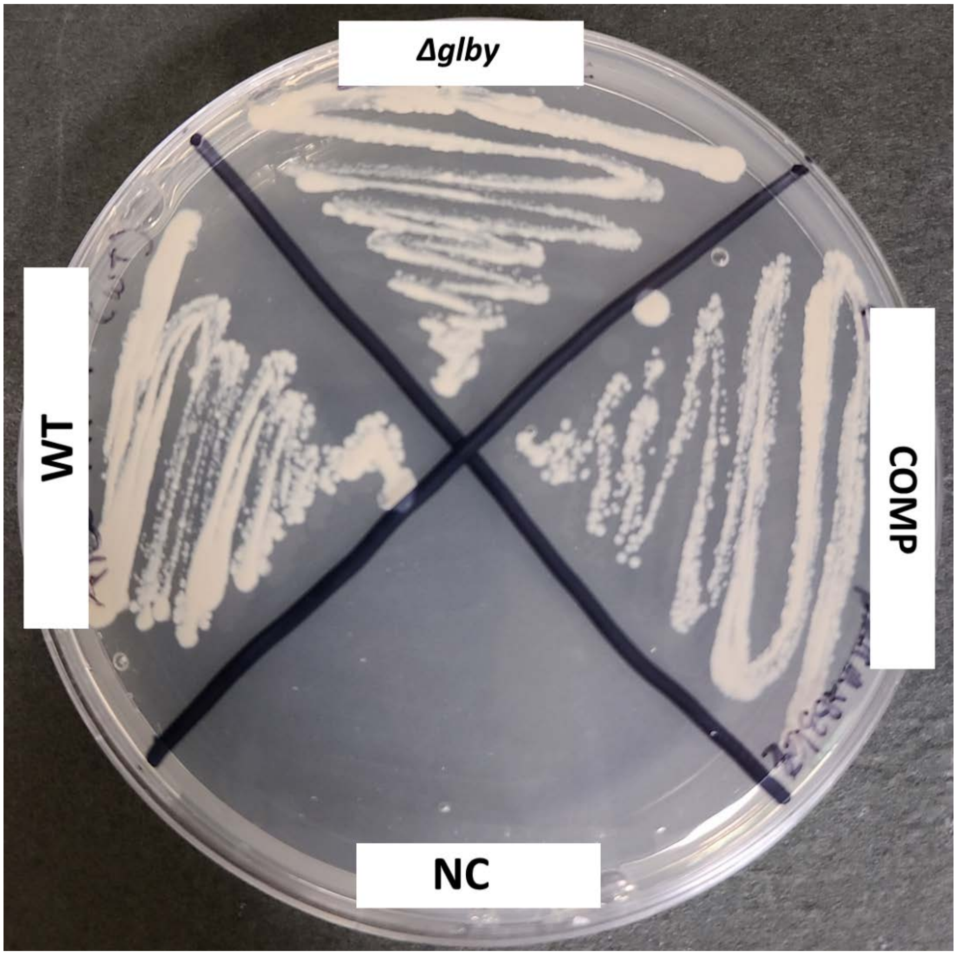

Figure S6. Colony morphology on 7H10 agar plates. Parent *Mab* strain ATCC 19977, *Δglby* and complemented strain (COMP). Region of agar that was not inoculated is designated as negative control (NC).

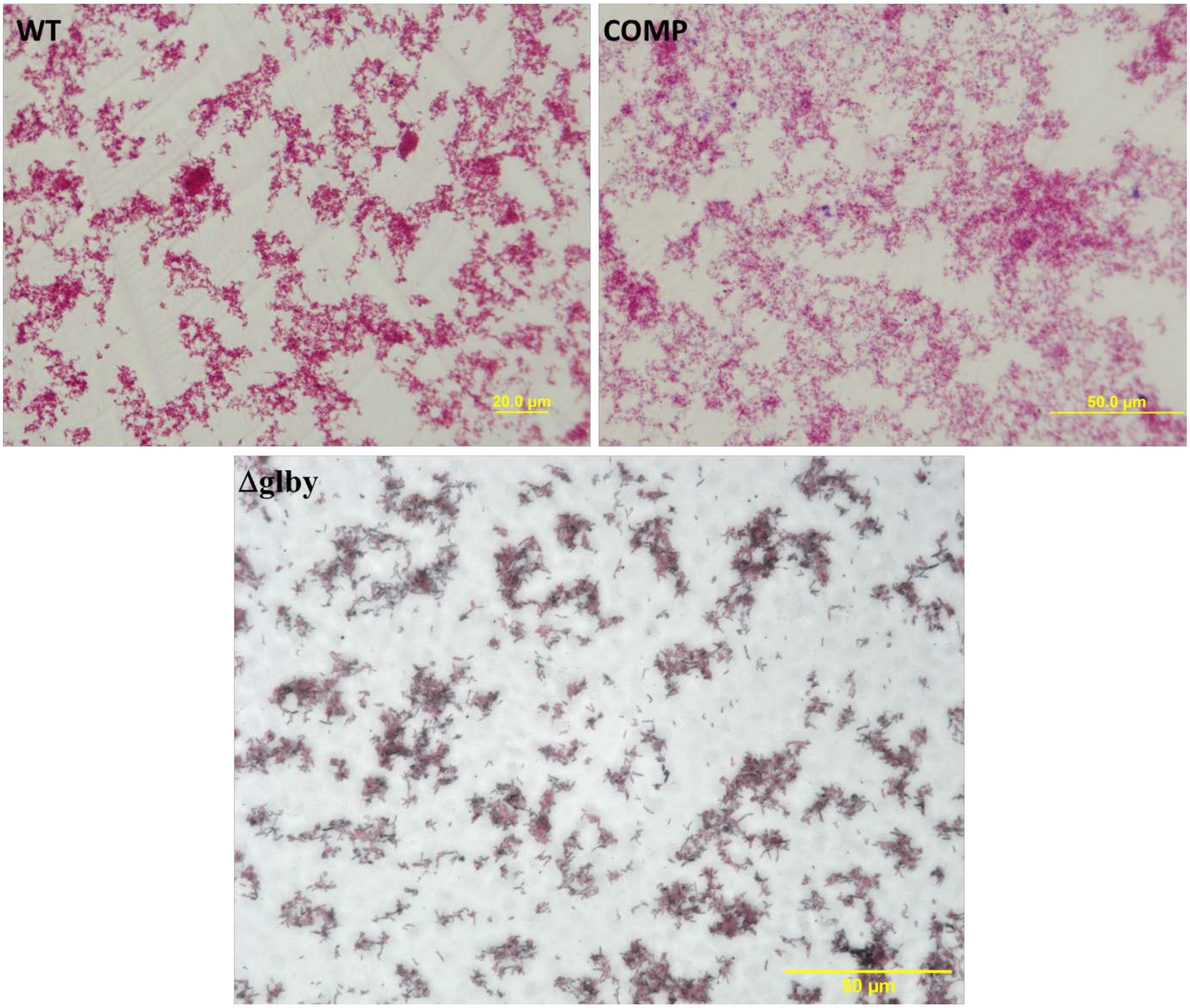

Figure S7. Ziehl-Neelsen stain of each strain at exponential phase. Each image was taken at 400x magnification. Parent strain *Mab* ATCC 19977 (WT), Δ*glby* and Complemented strain (COMP).

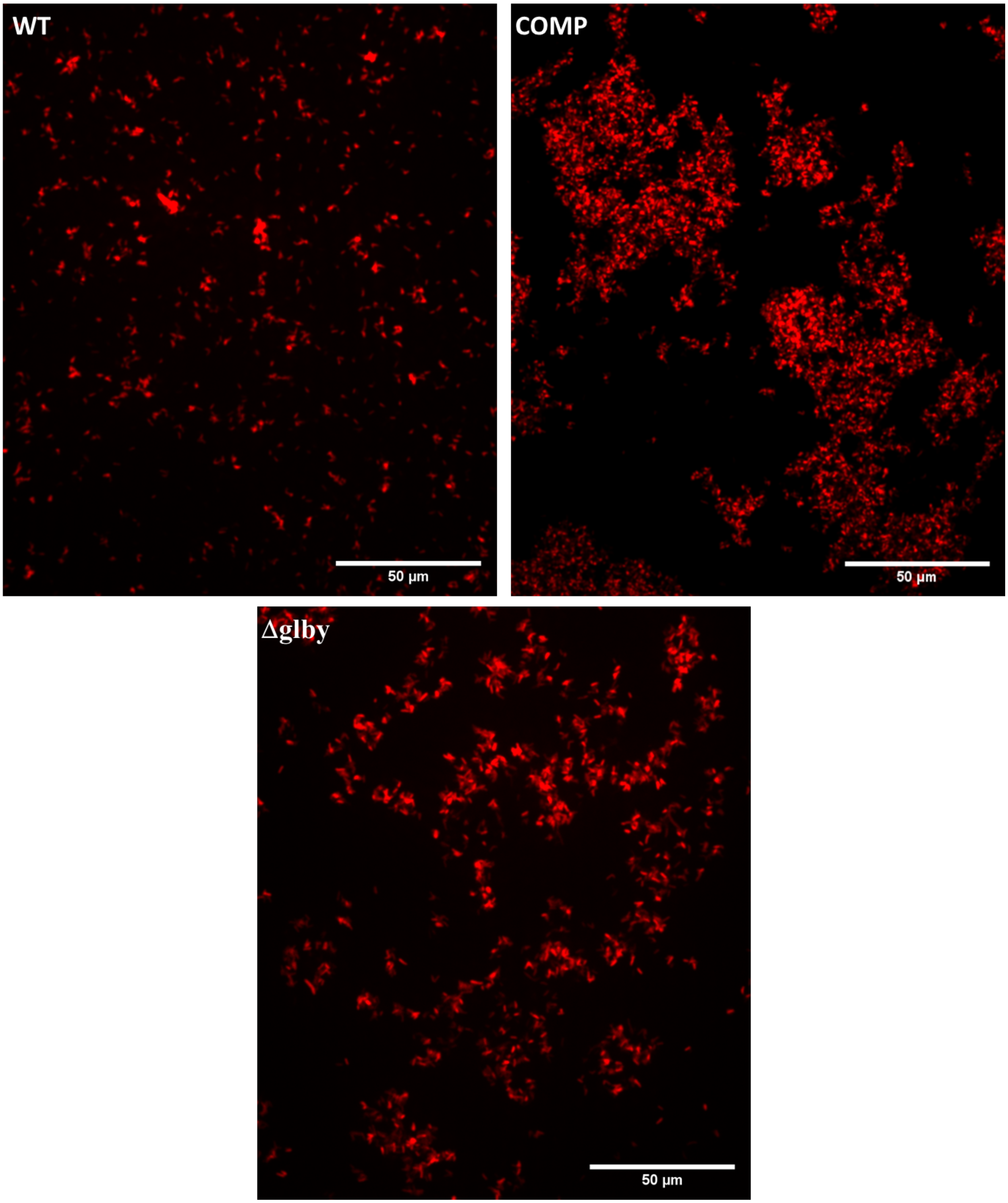

Figure S8. Auramine-Rhodamine stain of each strain at exponential phase. Each image was taken at 400x magnification. Parent strain *Mab* ATCC 19977 (WT), Δ*glby* and Complemented strain (COMP).

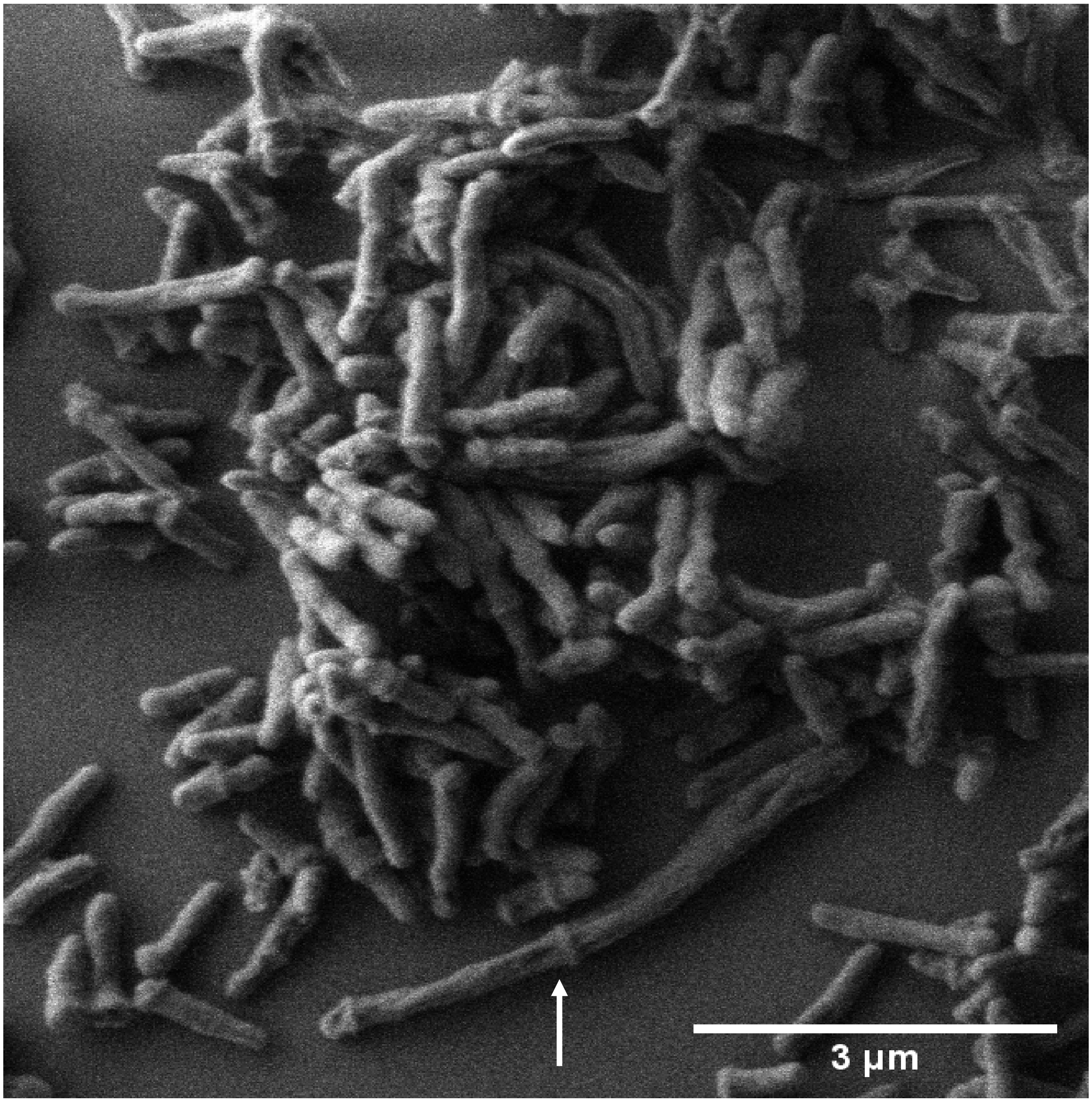

Figure S9. Scanning Electron Microscopy Image of *∆glby* cells in exponential phase. Arrow indicates the cell of 5.3µm in length.

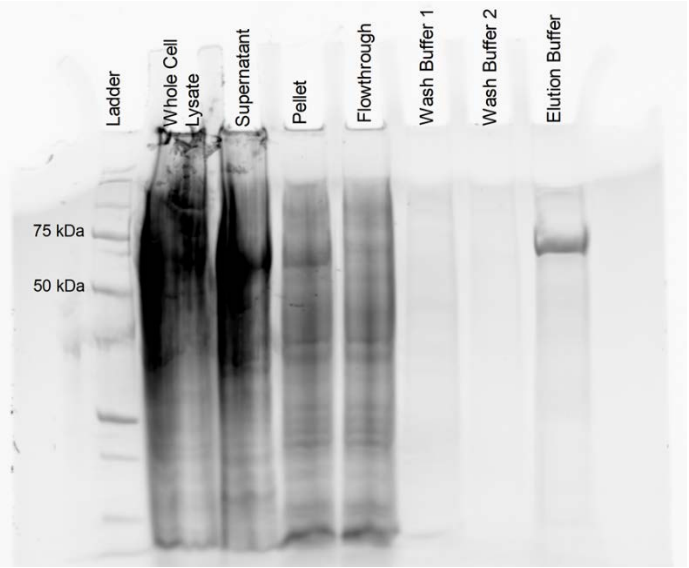

Figure S10. Various fractions during purification of Glby: Image of an SDS-PAGE of various fractions collected during the purification of Glby, expressed in *E. coli* DE3BL21 cells using pET28a+Glby, in a stain free gel. Equal volumes of each fraction were loaded in each lane to assess purity of Glby in the fraction in elution buffer.

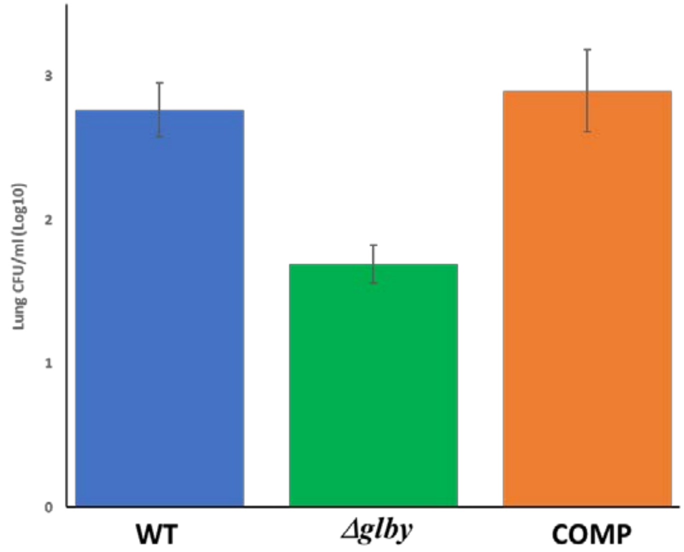

Figure S11. Pilot Study for implantation rate of strains in the lungs of mice. All mice were infected with aerosol of suspensions at OD (A_{600nm}=0.1) prepared from cultures of ATCC 19977 (WT), *Δglby* and complemented (COMP) strains at logarithmic phase of growth. Middlebrook 7H11 agar plates were inoculated with lung homogenates collected 24 hours after implantation and enumerated at 1 week of incubation at 37 ^{o}C.
